# Divergent symbiont communities determine the physiology and nutrition of a reef coral across a light-availability gradient

**DOI:** 10.1038/s41396-019-0570-1

**Published:** 2020-01-03

**Authors:** Christopher B. Wall, Mario Kaluhiokalani, Brian N. Popp, Megan J. Donahue, Ruth D. Gates

**Affiliations:** 1 Hawai‘i Institute of Marine Biology, University of Hawai‘i at Mānoa, PO Box 1346, Kāne‘ohe, HI 96744 USA; 2 Pacific Biosciences Research Center, University of Hawai‘i at Mānoa, 1993 East-West Road, Honolulu, HI 96822 USA; 3 Department of Earth Sciences, University of Hawai‘i at Mānoa, 1680 East-West Rd, POST 701, Honolulu, HI 96822 USA

**Keywords:** Stable isotope analysis, Biogeochemistry, Metabolism

## Abstract

Reef corals are mixotrophic organisms relying on symbiont-derived photoautotrophy and water column heterotrophy. Coral endosymbionts (Family: Symbiodiniaceae), while typically considered mutualists, display a range of species-specific and environmentally mediated opportunism in their interactions with coral hosts, potentially requiring corals to rely more on heterotrophy to avoid declines in performance. To test the influence of symbiont communities on coral physiology (tissue biomass, symbiont density, photopigmentation) and nutrition (δ^13^C, δ^15^N), we sampled *Montipora capitata* colonies dominated by a specialist symbiont *Cladocopium* spp. or a putative opportunist *Durusdinium glynnii* (hereafter, C- or D-colonies) from Kāne‘ohe Bay, Hawai‘i, across gradients in photosynthetically active radiation (PAR) during summer and winter. We report for the first time that isotope values of reef corals are influenced by Symbiodiniaceae communities, indicative of different autotrophic capacities among symbiont species. D-colonies had on average 56% higher symbiont densities, but lower photopigments per symbiont cell and consistently lower δ^13^C values in host and symbiont tissues; this pattern in isotope values is consistent with lower symbiont carbon assimilation and translocation to the host. Neither C- nor D-colonies showed signs of greater heterotrophy or nutritional plasticity; instead changes in δ^13^C values were driven by PAR availability and photoacclimation attributes that differed between symbiont communities. Together, these results reveal Symbiodiniaceae functional diversity produces distinct holobionts with different capacities for autotrophic nutrition, and energy tradeoffs from associating with opportunist symbionts are not met with increased heterotrophy.

## Introduction

Nutrient exchanges between scleractinian corals and dinoflagellate symbionts (Symbiodiniaceae, formerly *Symbiodinium* spp.) [1] underpin the success of reef-building corals as habitat engineers in coral reef ecosystems [2]. Reef corals are mixotrophic, reliant on the translocation of symbiont-derived compounds [3, 4] and heterotrophy in support of respiratory demands, calcification, and tissue growth [5–7]. In exchange, Symbiodiniaceae receive metabolic byproducts (i.e., CO_2_, NH_4_^+^) required for growth and photosynthesis [8]. However, reef corals are increasingly threatened by climate change and local stressors, which disrupt the coral-algae symbiosis and reduce ecosystem services provided by coral reefs [9, 10]. Associations with stress-tolerant symbionts can provide increased stress tolerance for corals albeit at the expense of coral performance [11], potentially requiring greater heterotrophic feeding to meet energy demands.

The genetic and functional diversity of Symbiodiniaceae shapes the energy balance and stress tolerance of reef corals. Taxonomic resolution of Symbiodiniaceae has been achieved using genetic markers, primarily the internal transcribed spacer 2 (ITS2) region of nrDNA [12, 13], and has revealed distinct symbiont genera and species (formerly clades and subclades) [1] with different capacities to support coral nutrition [14, 15] and tolerate environmental stress [16]. For instance, symbionts in the genus *Symbiodinium* (formerly clade A) and *Durusdinium* (formerly clade D) are common on shallow reef zones of the Red Sea and Caribbean [17, 18] and are also tolerant of high light and temperature stress. In particular, *Durusdinium* is observed in higher abundance on reefs impacted by local stressors or a history of bleaching [16, 19–21]. However, some members of *Symbiodinium* and *Durusdinium* have been identified as opportunistic generalist symbionts, assimilating and transferring less carbon and nitrogen compounds to their coral hosts compared to specialist symbionts with high host specificity, such as members of *Cladocopium* and *Breviolum* genera (formerly clade C and B, respectively) [14, 15, 17, 22, 23]. As a consequence, corals in association with stress-tolerant symbiont species may be less sensitive to environmental change but incur energetic tradeoffs [24], including reduced autotrophic nutrition, lower tissue, and skeletal growth rates, and attenuated reproductive output compared to more mutualistic symbionts (i.e., *Cladocopium* and *Breviolum*) [11, 25–29]. To cope with reductions in symbiont-derived nutrition from less-mutualistic symbionts, it has been hypothesized that coral hosts may shift towards greater heterotrophy to meet metabolic demands [30, 31], as has been observed in corals under experimental thermal stress ([32], but see *in situ* [33]) and on high turbidity reefs [34]. However, intraspecific changes in symbiont communities also reflect Symbiodiniaceae niche specialization, with some species being more efficient in uptake of carbon or nitrogen under low or high light and/or temperatures [17, 23]. Therefore, it is important to unravel the ecological contexts and effects of symbiont communities on coral nutrition to determine the capacity for corals to be nutritionally plastic or to cope with opportunistic symbiont effects on performance [17, 30].

Environmental factors, such as light availability [35–37], water quality [38], temperature [39], and bleaching history [40, 41] are important in shaping the composition of symbiont communities. Changes in photosynthetically active radiation (PAR, hereafter ‘light’) influence the ecological niche of reef corals [42], and many coral species exhibit shallow-to-deep transitions in symbionts in favor of those adapted to lower light levels. For example, shallow colonies of *Seriatopora hystrix* in Western Australia [43] are dominated by *Durusdinium*, whereas deeper colonies are more often dominated by *Cladocopium* symbionts. Similarly, shallow-to-deep transitions from *Symbiodinium* (shallow) to *Cladocopium* (deep) have been observed in *Stylophora pistillata* [17] in the Red Sea and *Orbicella faveolata* in the Caribbean [18]. Where depth and turbidity attenuate light, corals can rely on the photoacclimation potential of their endosymbionts [43] and/or heterotrophy to prevent energy deficits [34, 44].

Carbon and nitrogen stable isotope analyses of tissues have been applied to understand the balance of autotrophy and heterotrophy in reef corals and anemone model systems. These studies are widely interpreted as evidence for greater relative contributions of heterotrophy relative to autotrophy in corals during periods of resource limitation (i.e., reduced light availability) [45] or symbiosis disruption (i.e., coral bleaching) [46] and in regions characterized by high primary production [47]. However, the capacity for nutritional plasticity is not shared by all corals and depends on the biology of the coral host [48] and the optimization of skeletal morphology for light absorption or prey capture [49]. Recent research using corals and cnidarian model systems has also shown that the capacity for corals to increase heterotrophic feeding is also determined by Symbiodiniaceae communities [17, 30]. For instance, *Cladocopium*-associated *Stylophora pistillata* corals exhibited greater feeding rates but lower inorganic nitrogen assimilation rates than shallow *Symbiodinium*-associated colonies [17]. Similarly, *Durusdinium-*associated anemones did not shift towards greater prey capture when carbon translocation was low and exhibited lower feeding and digestion rates compared to anemones with *Symbiodinium minutum* symbionts [30]. These studies have shown symbiont community composition can influence the trophic ecology of corals and anemones with downstream implications for host performance. Due to uncertain boundaries in symbiont community distributions, few inquiries have examined the role of symbiont type on corals across natural environmental gradients (but see refs. [35, 43]) or symbiont-driven effects on coral feeding rates and the balance between autotrophic and heterotrophic nutrition. Yet, such inquires are necessary to understand the metabolic tradeoffs from distinct host-symbiont associations and symbiont effects on coral energy budgets.

Here, we examine changes in the physiology and heterotrophic capacity (carbon and nitrogen stable isotope analyses) of a Hawaiian reef coral (*Montipora capitata*) dominated by algal symbionts *Durusdinium glynnii* (ITS2: D1-4-6 [50]) or *Cladocopium* spp. (ITS2: dominated by C31 [51], but see ref. [52]) from <1–10 m during summer and winter. *M. capitata* shows depth-dependent shifts in symbiont communities (greater *Durusdinium* sp. at shallow depths [36]) and can exhibit stress-induced changes in nutritional modes [32]. Therefore, we predicted greater heterotrophic feeding in *M. capitata* under significant light attenuation [45, 47] and during association with opportunistic generalist symbionts (i.e., *Durusdinium*). We observed greater symbiont densities and lower carbon isotopic values indicative of lower rates of carbon fixation and autotrophy in corals associating with *Durusdinium*, but neither *Cladocopium-* nor *Durusdinium*-dominated colonies exhibited greater heterotrophic feeding across light gradients or seasons. Isotope values in corals across depth gradients have been previously explored ([47, 49]); however, our study offers the first evidence of symbiont community effects on host isotope values along a light gradient, where isotope values serve as proxies for coral productivity and nutritional modes. These findings reveal distinct traits of coral holobionts (symbiont and photopigment concentrations, carbon isotope values) that are determined by interactions between symbiont communities and environmental drivers, but do not lead to shifts toward greater heterotrophic nutrition.

## Materials and methods

### Site information

**Fig. 1:**
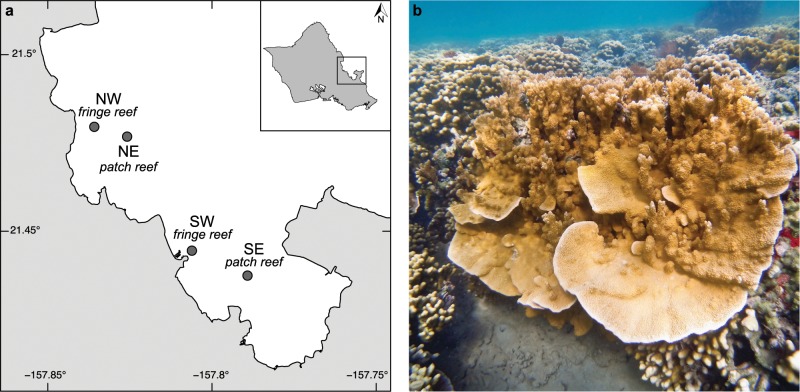
Site map and the coral *Montipora capitata*. **a** Map of Kāne‘ohe Bay on the windward side of O‘ahu, Hawai‘i and **b** the coral *Montipora capitata*. *Circles* represent locations where corals were collected. Northern Kāne‘ohe Bay locations were a fringe reef (‘Northwest’ [NW]: 21°28'46.5"N, 157°50'08.7"W) and a patch reef (‘Northeast’ [NE]: 21°28'36.5"N, 157°49'33.1"W); southern locations were a fringe reef (near He‘eia loko i‘a [fishpond)]) (‘Southwest’ [SW]: 21°26'40.3"N, 157°48'21.6"W) and the reefs of Moku o Lo‘e at the Hawai‘i Institute of Marine Biology (‘Southeast’ [SE]: 21°26'14.9"N, 157°47'21.3"W). (Photo credit: CB Wall).

*Montipora capitata* [53] colonies were sampled from four reefs spanning Kāne‘ohe Bay on the windward side of O‘ahu, Hawai‘i, USA (Fig. 1). Inshore Kāne‘ohe Bay is shallow (<15 m), and below ~6 m coral colonies are rare as fine silt dominates the benthos [54]. Sampling periods were defined as “summer” and “winter”, corresponding to historical periods of low and high seasonal rainfall [55]. Summer samples were collected in 2016 (June 8, July 11 and 29, August 3 and 9) [36], and winter samples were collected on December 19, 2016. Maximum seawater temperatures in 2016 for southern Kāne‘ohe Bay did not exceed historical averages (≤29 °C, [55]) and bleaching was not observed.

### Environmental conditions

Light environments across the four locations were described using cross-calibrated PAR light loggers (Odyssey, Dataflow Systems Limited, Christchurch, New Zealand) deployed at the four collection locations at 2 m-depth from 10 June 2016–11 January 2017 recording every 15 min and cleaned monthly. Integrated light-at-depth values for coral colonies was determined using light loggers at three depths (<1 m, 2 m, 8 m) 9–17 October 2016 and 9–19 November 2016 and calculating location-specific light attenuation coefficients (kd_x_) and daily light integrals (DLI), using a modified Beer-Lambert equation for light attenuation (see Supplemental Materials). Seawater temperatures were recorded at each site at 2 m-depth from June 2016 to January 2017 using cross-calibrated loggers (Hobo Pendant loggers ± 0.53 °C accuracy, Onset Computer Corp., Bourne, USA) recording at 15 min intervals.

Seawater dissolved nutrients and the isotope values of plankton (i.e., isotope end-members) were sampled 10 August and 19 December 2016 and used to account for site and seasonal differences in biogeochemistry. Seawater was collected at each location, filtered to remove plankton and debris (GF/F filters 0.7 μm nominal pore size), and frozen (−20 °C) in acid-washed (0.1 N HCl) HDPE bottles until analysis (see Supplemental Materials). Plankton/suspended particles were collected with vertical (<10 m) and horizontal plankton tows (63 μm mesh) and seawater (10 L) collected at 3 m-depth. Particles were size fractioned (<10, 10–100, 100–243 μm, and separate pooled samples of all particles <243 and >243 μm), filtered onto GF/F filters (0.7 μm), and dried (60 °C) until isotope analyses (detailed below).

### Coral sampling and tissue analysis

At each of the four reefs in summer and winter, branch tips (4 cm^2^) were collected from five *M. capitata* colonies at each reef location within three depth strata (0–2 m, 2–5 m, 5–10 m) spanning the depth gradient where colonies were observed (*n* *=* 15 samples per location). Colony depth and time of collection was noted to correct for tidal height, with final depths corrected to mean seawater height using NOAA tide data at 6-min intervals for Moku o Lo‘e (Station ID: 1612480) from CO-OPS API in a custom R code [36]. Immediately after collection, corals were flash-frozen in liquid nitrogen, transported to HIMB, and stored at −80 °C.

Coral tissues were removed from the skeleton using an airbrush with filtered seawater (0.7 μm). The tissue slurry was homogenized and aliquots taken for physiology and isotopic analysis. Symbiont cell densities were determined by microscopy (*n* *=* 4–8 counts) using a haemocytometer. Photopigments (chlorophyll *a* and *c*_*2*_) were extracted in 100% acetone and measured by spectrophotometry [56] (see Supplemental Materials). Ash-free dry weight (AFDW) (i.e., total host + symbiont biomass) of holobiont tissues was quantified as the difference between dried (60 °C, 24 h) and combusted masses (450 °C, 4 h). All physiological metrics (cell densities, chlorophyll concentrations, total biomass) were standardized to coral surface area, determined using the wax-dipping technique [57]; chlorophylls were additionally normalized to symbiont cell abundance [58].

### Stable isotope analysis

Stable isotope analyses were performed on suspended particles and plankton from filtered seawater (detailed above), separated coral and symbiont tissues, and coral skeleton material to examine the trophic ecology and nutrient exchanges between host and symbiont. Samples were not acidified [59] or bleached [60] prior to isotope analysis. Coral and symbiont tissues were separated by centrifugation with filtered seawater rinses [45] and filtered to remove skeletal debris and avoid the contribution of carbonates on isotope values [61]. The effectiveness of filtration was confirmed by regressing sample C:N against δ^13^C values (see Supplemental Materials). Carbon (δ^13^C) and nitrogen (δ^15^N) and tissue molar C:N ratio for coral host (δ^13^C_H_, δ^15^N_H_, C:N_H_) and algal symbiont (δ^13^C_S_, δ^15^N_S_, C:N_S_) tissues were determined using elemental combustion-isotope ratio mass spectrometry (IRMS) with glycine standards characterized by international reference materials and in-house standards of tuna white muscle tissue, with <0.2‰ sample analytical precision and accuracy (see Supplemental Materials). The relative differences of host and symbiont carbon (δ^13^C_H-S_) and nitrogen (δ^15^N_H-S_) isotope values were calculated as metrics for heterotrophic capacity (i.e., δ^13^C_H-S_) and changes in trophic enrichment (i.e., δ^15^N_H-S_) [46, 62].

Skeletal carbon (δ^13^C_Sk_) and oxygen (δ^18^O) isotope values in the uppermost layers of the coral skeleton (ca.1-2 mm, ca. 80 μg) were measured by acidifying skeletal material (100% orthophosphoric acid) under vacuum (90 °C) and measuring released CO_2_ by IRMS; reference material (marble CaCO_3_ standard) deviated by 0.02‰ (δ^18^O) and <0.2‰ (δ^13^C). Kinetic isotope effects (KIE) on skeletal carbonates were investigated using estimates for carbon and oxygen isotope equilibrium (δ^13^C_eq_ and δ^18^O_eq_, respectively) for skeletal aragonite, estimated using values from Schoepf et al. [63] (see Supplemental Materials). Stable isotope ratios are reported using delta values (δ) in permil (‰) notation relative to Vienna Pee-Dee Belemnite (V-PBD) (δ^13^C, δ^18^O) and atmospheric N_2_ (air) (δ^15^N).

### DNA extraction and symbiont community analysis

Numerous reports have shown *M. capitata* Symbiodiniaceae communities in Hawai‘i to be comprised only of *Cladocopium* and *Durusdinium*, with *Durusdinium* found only in Kāne‘ohe Bay [20, 51]. We quantified Symbiodiniaceae communities by extracting symbiont DNA (following [51]) and using qPCR targeting actin gene loci for *Cladocopium* [51] and *Durusdinium* [50]. The relative abundance of *Cladocopium* and/or *Durusdinium* was determined for each colony using two replicate qPCR reactions, with normalization applied for reporter dye fluorescence intensity with estimates of DNA extraction efficiency and actin gene copy number for each symbiont species (detailed in, [64]). Each colony was categorized as either *Cladocopium*- or *Durusdinium*-dominated (i.e., C- and D-colonies) based on the numerical abundance of these two genera (threshold: proportion > 0.5) [36] (for further information see Supplemental Materials).

### Statistical analysis

Differences in daily light integral were analyzed in a linear mixed effect model with location and season as fixed effects and date-of-collection as a random effect. Other environmental data (dissolved nutrients, plankton) were analyzed in a linear model with reef locations and seasons as fixed effects. The dominant Symbiodiniaceae species in *M. capitata* (i.e., categorical with symbiont dominance [C or D] as qPCR > 0.5 proportion) was analyzed using a logistic model with a binomial distribution; colony depth, season, and location were treated as main effects [36]. Models were tested using AIC model selection [65] and likelihood ratio tests of main effects. Tests of the relationship between response metrics, spatiotemporal factors (i.e., season, location, colony depth-bins), and symbiont community were performed using a principal components analyses (PCA) of a scaled and centered correlation matrix; PERMANOVA tests of main effects were evaluated in the package *vegan* [66]. Biological response variables (total biomass, symbiont densities, areal and symbiont cell-specific chlorophyll concentrations) were analyzed in a linear mixed effect model (*lme4* [67]) with season (winter *vs*. summer), light-at-depth (continuous variable), and dominant symbiont community (*Cladocopium vs*. *Durusdinium*) as fixed categorical effects and location as a random effect; final models were selected according to AIC. Pairwise post-hoc slice-tests of main effects were performed using estimated marginal means (*emmeans* [68]). Analysis of variance tables were generated using *car* [69] and mixed-effect *lmerTest* packages [70]. Subsequently to testing main effects on response variables, linear regressions were used to evaluate the influence of symbiont traits (cell density, total areal chlorophyll, chlorophyll symbiont cell^−1^) on δ^13^C values in the host and symbiont in each season. All statistical analyses were performed in R version 3.5.2 [71]. Data and scripts to reproduce analyses and figures are archived at Zenodo [72] and available at Github.

## Results

### Environmental conditions

Light availability, expressed as the daily light integral (i.e., DLI), was higher in summer relative to winter (*p* *<* 0.001) and similar across all locations except for lower DLI at the southwest location (*p* *<* 0.001) (Table S1, Fig. S1a). Estimates for DLI from June 2016 to January 2017 (mean ± SE, *n* *=* 163–198) at <1 m were 17.3–21.3 ± 0.6 mol photons m^−2^ d^−1^ at all locations, except the southwest location (9.8 ± 0.5 mol photons m^−2^ d^−1^) (Fig. S2). Relative to <1 m, DLI was attenuated by 61 and 82% at 2 m and 8 m-depths, respectively. Seasonally averaged DLI from loggers at 2 m were 9.3–15.6 mol photons m^−2^ d^−1^ (summer) and 2.8–8.4 mol photons m^−2^ d^−1^ (winter). Daily mean seawater temperatures were similar across all sites, with summer maximum temperatures ranging from 27 to 29 °C; winter temperatures ranged from 24 to 25 °C (Fig. S1b). Nutrient concentrations remained low and changes among sites and seasons were small (Fig. S3). Phosphate, ammonium, and nitrate + nitrite (N + N) concentrations were higher in winter compared to summer (*p* *≤* 0.046) (Table S1), and the two northern sites had higher N + N (summer and winter) and phosphate (winter only) concentrations compared to southern sites (*p* *<* 0.001).

Carbon and nitrogen isotope values of suspended particles and plankton did not differ between locations (*p* *≥* 0.146) and seasonal effects on δ^15^N values were small (0.3‰ difference) (*p* *≥* 0.049) (Table S1). The mass of organic fractions (i.e., AFDW) represented in suspended materials (0.7 to <243 μm) were also found to be similar across sites and seasons at ca. 1–2 mg L^−1^ (data not shown). Therefore, isotope values were pooled among the locations and seasons to generate isotope end member plots (Fig. S4). Mean δ^13^C values were similar for all size fractions across seasons and locations (−18.1 to −21.1‰). Mean δ^15^N values were lowest in <10 μm (5.3‰), intermediate in 100–243 μm (6.5‰), and highest in 10–100 μm fractions (7.4‰). In pooled samples, small particles (0.7 to <243 μm) were ca. 1‰ depleted in ^15^N relative to large particles (>243 μm) (5.9 and 6.8‰, respectively).

### Symbiont community

**Fig. 2:**
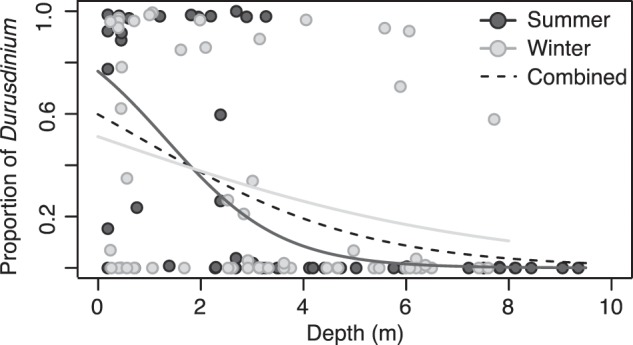
Depth distribution of symbiont communities. Symbiont community in *Montipora capitata* colonies collected in summer (*dark gray*) and winter (*light gray*) as a function of the proportion of *Durusdinium* relative to *Cladocopium* across collection depths. Lines represent logistic regression models by each season (*solid* lines) and a pooled dataset (*dotted* line).

Corals were collected over comparable depth ranges in summer (0.2–9.4 m) and winter (0.2–7.7 m) (Fig. S5). The distribution of dominant Symbiodiniaceae was depth-dependent in both seasons (*p* *<* 0.001) and did not differ among locations (*p* *=* 0.339). Importantly, because colonies were randomly sampled in each season (i.e., individuals were not tagged and resampled), these data cannot be used to evaluate symbiont shuffling across time, but instead reflect ecological patterns of holobionts dominated by symbionts within each period. We observed a greater number of D-colonies in the shallows, and C-colonies increased with depth, although a sharper decrease in D-colonies with depth was observed in summer (*p* *=* 0.013) (Fig. 2). D-colonies were observed from 0.4–3.3 m-depth (summer) but occasionally deeper (7.7 m, winter), although at low frequencies (Fig. 2). *Durusdinium* was also observed as a background symbiont member at community proportions of <1–35% (mean proportion 9%) across depths in summer (0.8–7.8 m) and winter (0.2–6.5 m).

### Principal component analysis of biological responses

**Fig. 3:**
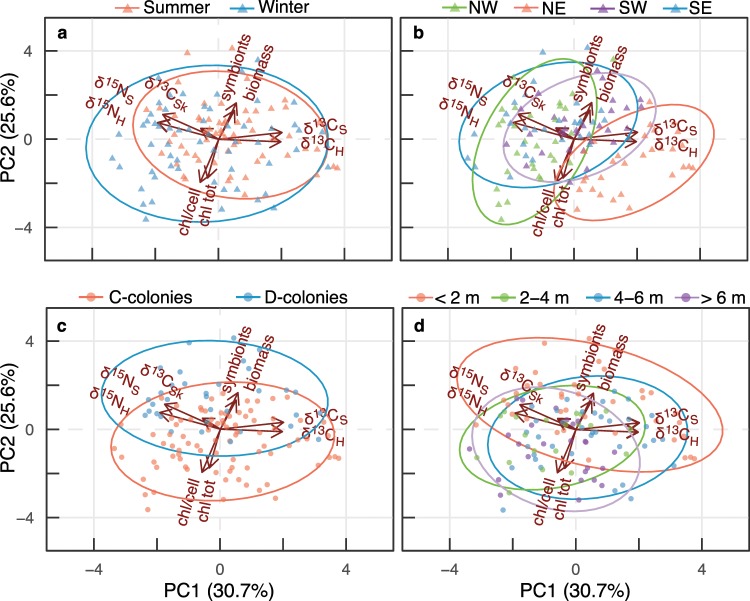
Multivariate analyses of coral traits. Principal component analyses (PCA) on a matrix of physiological responses and isotope values in the coral *Montipora capitata* evaluating the influence of **a** season, **b** location, **c** symbiont community, and **d** depth-bin. Axis values in parentheses represent the proportion of total variance associated with the respective PC. Arrows represent significant (*p* < 0.05) correlation vectors for response variables; ellipses represent 90% point densities. See Table 1 for response metric details.

Two principal components (PCs) explained 56% of the variance in coral physiological and isotope responses among seasons, locations, symbiont communities, and colony depth-bins (at 2 m-intervals) (Fig. 3). PERMANOVA results showed biological responses were most influenced by symbiont community (*p* *<* 0.001) followed by location (*p* = 0.003) and season (*p* = 0.009), whereas colony depth-bins were non-significant (*p* *=* 0.063). Overall, PC1 (30.7% variation) separates corals with higher tissue δ^13^C values from corals with high δ^15^N values, and PC2 (25.6% variation) separates corals with high tissue biomass and symbiont density from those with high chlorophyll. Seasonal effects on coral responses were similar; however, greater shifts in chlorophylls and nitrogen isotope values were observed in the winter compared to the summer (Fig. 3a). Sampling locations did not produce distinct groups with the exception of the northeast location, which had higher PC1 values associated with δ^13^C values (Fig. 3b). Symbiont communities were distinguished along PC2 with D-colonies being associated with high symbiont densities and coral biomass and C-colonies having greater chlorophylls (total areal concentrations and per symbiont cell) (Fig. 3c). Zonation across depths showed corals at <2 m-depth were most distinct from other depths, and this mirrored the effect of symbiont community, particularly the positive correlation with PC1 and δ^13^C values (Fig. 3d). In addition, there was less variation between corals with increasing depth indicated by reduced ellipse area in deeper colonies relative to those at the surface.

### Physiology and isotope measurements

**Fig. 4:**
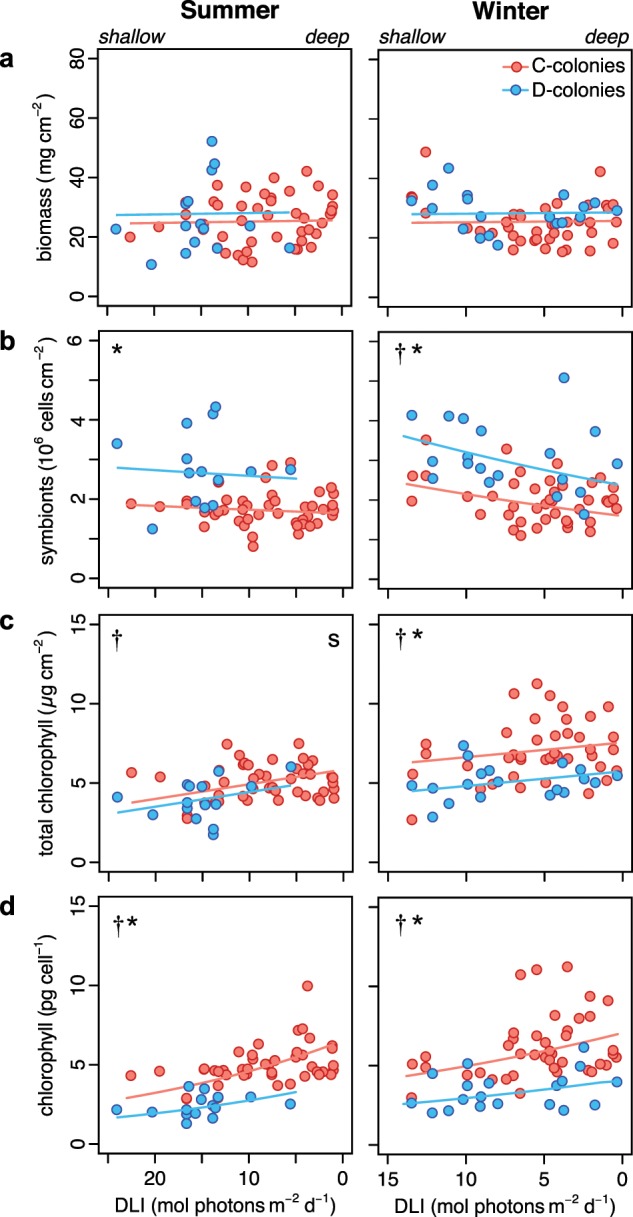
Physiological traits. Physiological metrics for *Montipora capitata* colonies dominated by C (*Cladocopium*) or D (*Durusdinium*) symbionts collected from four Kāne‘ohe Bay reef locations in summer (left) and winter (right) spanning a light availability gradient across <1– 10 m-depth. Area-normalized **a** total tissue biomass, **b** symbiont cell densities, **c** total chlorophyll (*a* *+* *c*_*2*_), and **d** chlorophyll per symbiont cell. Solid lines represent the linear mixed effect model fits. *Symbols* indicate significant differences (*p* < 0.05) between symbiont communities (*), in response to light (†), and between seasons (*s*).

Total biomass did not vary as a function of season, light, or symbiont community (*p* *≥* 0.104) (Table 1, S2, Fig. 4a). Symbiont densities (cells cm^−2^) were 56% higher in D-colonies relative to C-colonies (*p* *<* 0.001) and increased with light availability for all colonies (*p* *=* 0.010), and this effect was most pronounced in winter (*p* *=* 0.004) (Fig. 4b). Total chlorophyll (μg *a* *+* *c*_2_ cm^−2^) was higher in winter (*p* < 0.001) and increased as light declined (*p* *=* 0.004) (Table 1). Chlorophyll concentrations were equivalent between C- and D-colonies in the summer but were 26% higher in C-colonies during winter months (*p* *=* 0.022) (Fig. 4c). Chlorophyll (*a* + *c*_2_) per symbiont cell (pg cell^−1^) did not differ between seasons (*p* *=* 0.096), but increased as light declined (*p* *<* 0.001) and was 46% higher in C-colonies relative to D-colonies (*p* *<* 0.001) (Table 1, Fig. 4d). As a random effect, location accounted for 9–32% model variance for physiological responses (Fig. S6).

**Table 1:** Statistical analysis of *Montipora capitata* physiology and tissue isotope values from four locations in Kāne‘ohe Bay along a light-availability gradient in summer and winter.

*Response variable*	*Effects*				
	Season	Light	Symbiont	Season × Light	Season × Symbiont
Biomass	–	–	–	–	–
Symbionts	–	0.010	<0.001	0.004	–
Total chlorophyll	<0.001	0.004	<0.001	–	0.022
Chlorophyll per cell	–	<0.001	<0.001	–	–
δ^13^C_H_	–	<0.001	<0.001	–	0.031
δ^13^C_S_	–	<0.001	<0.001	–	0.001
δ^13^C_H-S_	0.002	–	<0.001	0.040	0.037
δ^13^C_Sk_	0.009	–	–	–	–
δ^15^N_H_	–	0.040	–	–	–
δ^15^N_S_	–	–	0.008	–	0.017
δ^15^N_H-S_	–	0.018	0.002	–	<0.001
C:N_H_	–	–	–	–	–
C:N_S_	–	–	–	–	–

Table information shows significant model effects (*p* < 0.05); dashed lines indicate no significant effects (*p* > 0.05). Subscripts indicate either host (H) or symbiont (S) tissues, or their relative difference (H-S), and skeletal carbonates (Sk).

*Season* summer or winter; *Light*  light at depth of collection; *Symbiont* either *Cladocopium* (formerly clade C) or *Durusdinium* (formerly clade D) dominated symbiont community

**Fig. 5:**
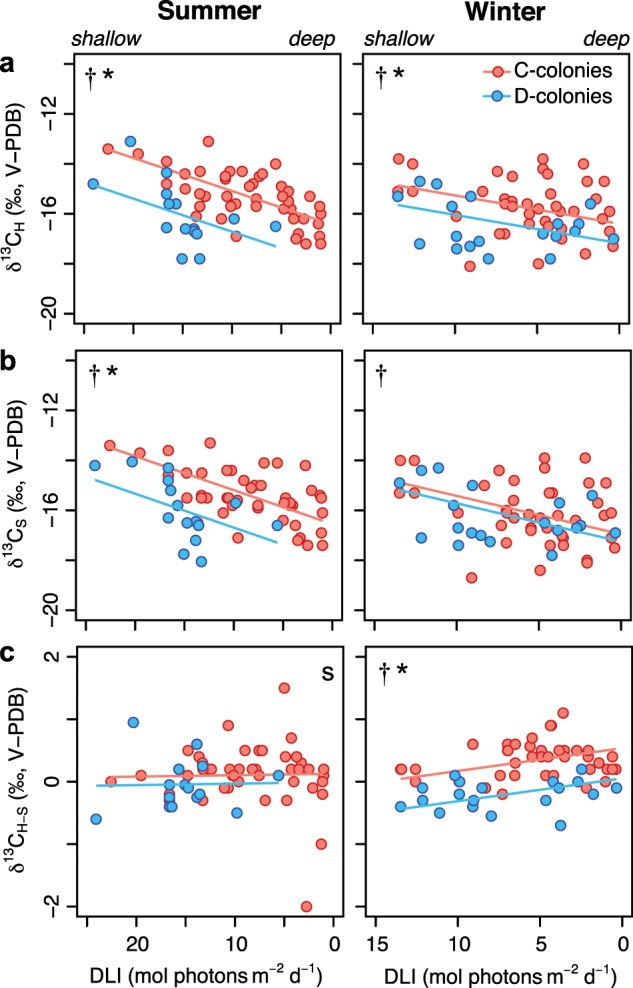
Stable isotope values. Carbon stable isotope values for *Montipora capitata* colonies dominated by C (*Cladocopium*) or D (*Durusdinium*) symbionts collected from four Kāne‘ohe Bay reef locations in summer (left) and winter (right) spanning a light availability gradient across <1– 10 m-depth. Values are for **a** coral host (δ^13^C_H_) **b** symbiont algae (δ^13^C_S_) and **c** their relative difference (δ^13^C_H-S_) in permil (‰) relative to carbon standards (Vienna Pee Dee Belemnite: V-PDB). Solid lines represent linear mixed effect model fits. *Symbols* indicate significant differences (*p* < 0.05) between symbiont communities (*), in response to light (†), and between seasons (*s*).

The carbon isotope composition of both coral host (δ^13^C_H_) and the symbiont algae (δ^13^C_S_) became progressively ^13^C-depleted (i.e., lower δ^13^C values) as light availability declined (*p* *<* 0.001) (Table 1, S3, Fig. 5a, b). Host and symbiont δ^13^C values in C-colonies were ^13^C-enriched (i.e., higher δ^13^C values) relative to D-colonies (*p* *<* 0.001), particularly in the summer (*p* *≤* 0.031). Host δ^13^C values were 1.6‰ and 0.8‰ higher in C-colonies relative to D-colonies in summer and winter, respectively (Fig. 5a), whereas symbiont δ^13^C values were 1.5‰ higher in C-colonies in summer but comparable between both C- and D-colonies in winter (Fig. 5b). The difference in host and symbiont carbon isotope values (δ^13^C_H-S_) differed between C- and D-colonies (*p* *<* 0.001) (0.3‰), and this was driven by greater differences in δ^13^C_H-S_ during the winter. In winter, δ^13^C_H-S_ increased as light decreased with depth (*p* *=* 0.040) and was lower in D-colonies (*p* = 0.037) (Table 1, S3, Fig. 5c). Skeletal carbonate δ^13^C values (i.e., δ^13^C_Sk_) varied across samples, but the range (−4 to −0.4‰) and average δ^13^C_Sk_ values (ca. −2.5‰) were consistent among seasons (<0.5‰ change) and did not systematically decline under low light (*p* *=* 0.639) (Fig. S7). Location accounted for 17–27% of all carbon isotope model variances (Fig. S6).

Tests of the influence of symbiont traits on isotope values (Fig. S8a–c) revealed δ^13^C_S_ values increased with symbiont density in the winter, but not summer, for C-colonies (*p* *<* 0.001, *R*^2^ = 0.360) and D-colonies (*p* = 0.007, *R*^2^ = 0.708). δ^13^C_S_ values were not related to changes in total chlorophyll in either season for C- or D-colonies (*p* *≥* 0.414). In C-colonies alone, δ^13^C_S_ values increased as chlorophyll cell^−1^ declined in summer (*p* = 0.004, *R*^2^ = 0.173) and winter (*p* = 0.030, R^2^ = 0.115). δ^13^C_H_ values for C- and D-colonies exhibited similar effects as observed for δ^13^C_S_ (data not shown). δ^13^C_H-S_ values were only influenced by symbiont densities and became more positive as symbiont densities declined in summer D-colonies (*p* = 0.004, *R*^2^ = 0.478) and in both winter C-colonies (*p* = 0.014, *R*^2^ = 0.145) and D-colonies (*p* = 0.007, *R*^2^ = 0.361) (Fig. S9).

Nitrogen isotope values of the coral host and symbiont did not show meaningful change across seasons, light availabilities, or symbiont communities (Table 1, S3, Fig. S10) (see Supplemental Materials). Molar ratios of carbon:nitrogen (C:N) in host and symbionts showed no significant effects (*p* *≥* 0.134) (Table 1, S3, Fig. S11). Location explained a large portion of variance for δ^15^N_H_ (75%) and δ^15^N_S_ (80%) models but less (<20%) in δ^15^N_H-S_ and C:N models (Fig. S6).

## Discussion

Symbiodiniaceae diversity, function, and community physiological traits have a clear influence on coral stable isotope values and these effects manifested across a narrow depth gradient in relation to rapid light attenuation. Species-specific symbiont attributes, including higher symbiont densities, lower chlorophylls per cell, and lower δ^13^C values indicate lower rates of carbon assimilation/translocation and potential host exploitation by *Durusdinium glynnii*. However, neither environmental nor symbiont community effects resulted in greater heterotrophic nutrition, suggesting photoacclimation may be central to coping with energetic tradeoffs incurred by corals in low-light environments as well as in association with opportunistic symbionts, such as *D. glynnii*. Finally, we identify symbiont community function as an important, yet often overlooked, component to isotopic investigations into coral physiological ecology.

Light attenuation in Kāne‘ohe Bay was rapid across a narrow depth gradient with maximum PAR at 1 m-depth across all sites (ca. 1400–2300 μmol photons m^−2^ s^−1^) attenuated to 50–350 μmol photons m^−2^ s^−1^ at 8 m-depth—equivalent to PAR attenuation observed at 40–70 m in coral reefs of the Red Sea [73] and 20–40 m in the Caribbean [74, 75]. Therefore, depth-dependent changes in light availability is a principal force acting to partition *M. capitata* symbiont communities with a greater frequency of *Durusdinium* observed at shallow depths across seasons and reef locations. This depth-dependent distribution in response to reduced light availability can in part be explained by close proximities to multiple watersheds and high concentrations of fine-grained particles that dominate inshore Kāne‘ohe Bay reefs [54], which settle slowly and are easily re-suspended, creating significant and persistent light attenuation [76].

Globally, the prevalence of *Durusdinium* is higher on human-impacted reefs, including those experiencing high temperatures and/or recent thermal stress and high rates of sedimentation [77]. Therefore, the shallow-water dominance of *D. glynnii* in Kāne‘ohe Bay *M. capitata* suggests a greater capacity for this species to tolerate a combination of more frequent temperature anomalies [20, 51] and high irradiances [35] compared to *Cladocopium* spp. In Hawai‘i, reports of *Durusdinium* have been limited to *M. capitata* from Kāne‘ohe Bay, which has a history of persistent human impacts (dredging, sewage outflow) and high frequency of thermal stress anomalies [20, 78]. This combination of human impacts and thermal stress anomalies may have allowed a niche for *Durusdinium* to exploit, while also driving local adaptation in the coral hosts for increased tolerance to thermal stress [79]. While *Durusdinium* prevalence in shallow depths (ca. < 3 m) indicates a tolerance to elevated light and temperature, the rarity of D-colonies at depth also indicate Symbiodiniaceae niche partitioning in response to light availability and perhaps a limited capacity for *Durusdinium* to adapt to low PAR or in response to changing light spectra [73, 80]. Intraspecific shifts in symbiont communities generally occur at deeper depths, for instance *Stylophora pistillata* transitions from *Symbiodinium microadriaticum* (ITS2: A1) (<10 m) to *Cladocopium* spp. (>40 m) in the Red Sea [17], and *Seriatopora hystrix* transitions from *Durusdinium* (<23 m) to *Cladocopium* (>23 m) in western Australia [35]. However, high turbidity in Kāne‘ohe Bay has resulted in *M. capitata* symbiont community depth zonation to within a few meters (<2 m) of the surface ([36]; this study).

D-colonies had lower δ^13^C values, indicating greater isotope fractionation and/or lower rates of carbon assimilation and translocation to the coral host compared to C-colonies [14, 15]. While δ^13^C values are also influence by tissue properties, sources of inorganic carbon, and the contribution (and composition) of respiration CO_2_ to the internal carbon pool [81], patterns observed here provide indirect evidence consistent with lower rates of photosynthesis and carbon fixation. *M. capitata* did not show signs of changes in heterotrophic feeding in response to changing light conditions, seasons, or due to hosting different symbiont species. However, substantial and persistent effects of symbiont community on isotope values were observed, which in conjunction with physiological attributes of the holobionts, reveal differing capacities of *Cladocopium* spp. and *D. glynnii* to fix and translocate carbon to their hosts. Photoacclimation had a clear influence on *M. capitata* host and symbiont δ^13^C values, indicated by a positive relationship with symbiont densities in winter and a negative relationship with photopigmentation (pg cell^−1^) (Fig. S8), and these effects were more pronounced in C-colonies.

Changes in symbiont densities and photopigmentation are important photoacclimation responses to maximize light-use efficiency in reef corals [42] and influence holobiont energetics, calcification [29], and sensitivity to thermal stress [82]. Changes in chlorophylls, however, are but one mechanism corals employ to optimize light capture and attenuate damage from photoinhibition. Photoacclimation in shallow corals can also include increased concentrations in photoprotective pigments of the symbiont (xanthophyll, β-carotene) and host (fluorescent proteins), or greater dissipation of absorbed light-energy through non-photochemical quenching (i.e., NPQ) [83]. Similar mechanisms of modulating pigmentation and light-energy dissipation are also used by corals in deep-water habitats acclimated to low (and variable) light intensity of different spectral compositions. In the case of the latter, host-derived photoconvertible red fluorescent proteins (pcRFPs) appear to contribute to low-light adaptation in corals through the transformation of blue-to-orange light wavelengths that benefit symbiont light capture in deep-water corals [84]. Such changes in host pigmentation have not been tested in *M. capitata* but may be of importance considering this species exhibits depth-dependent distributions of two distinct color morphs (orange *vs*. brown) [36].

We observed D-colonies to have 54–58% greater symbiont densities and 0.8–1.6‰ lower δ^13^C values compared to C-colonies; however, C-colonies had 46% higher chlorophyll cell^−1^ and showed greater potential to regulate areal and symbiont cell-specific chlorophyll concentrations in response to changing environmental conditions. While Symbiodiniaceae cell size can influence symbiont densities, the coccoid cell sizes for other *Durusdinium* and *Cladocopium* species overlap [1, 50]. High symbiont densities increase symbiont self-shading, which can reduce internal light values by 90% relative to those at the colony surface [85] and cause declines in net photosynthesis through light and/or carbon limitations. In both cases, reductions in photosynthesis rates (inferred here by lower δ^13^C values) reduce nutritional benefits to the host [82]. Changes in photosynthesis relative to respiration (P:R) can also be influenced by symbiont communities and environmental contexts, where metabolic *savings* from lower respiration rates with increasing depth (higher P:R) transition to metabolic *costs* (lower P:R) in symbionts unable to acclimate to light limitations [35]. Therefore, the clear differences in symbiont traits and isotope values between C- and D-colonies provide evidence for different physiological and biochemical processes in these two holobionts. In addition, the greater ability for C-colonies to photoacclimate in response to changing light-availability indicate light as a driver in the niche partitioning of these symbiont species.

Differences in symbiont densities can also be influenced by Symbiodiniaceae growth rates and/or responses to nutrient availability [86]. For example, symbiont-derived photosynthates stimulate the recycling of ammonium waste by the host and nitrogen incorporation into amino acids, serving as a negative-feedback loop regulating symbiont densities by limiting nitrogen to the symbiont [87]. Paradoxically, reduced symbiont photosynthesis increases nitrogen availability to the symbiont, and as a result, symbiont biomass and densities may increase. Similarly, corals exposed to sub-bleaching thermal stress experience reduced carbon transferred from their symbionts, but symbiont carbon and nitrogen assimilation and growth increases [18]. Symbiont community effects on host, in particular through effects on ammonium metabolism, may also influence nitrogen availability and increase symbiont growth and densities [31]. For instance, *Exaiptasia pallida* anemones infected with *Durusdinium trenchii* increased the transport of ^15^N-labeled products through the urea cycle and had greater glutamine pool enrichment (a primary enzyme in the assimilation of ammonium) compared to *Breviolum minutum*-hosting anemones, which did not show urea cycle feedback but had greater glutamine synthetase enzyme abundance [31, 88]. Such effects on host metabolism dictated by Symbiodiniaceae community may serve to regulate nitrogen availability and symbiont densities and should be further tested in reef corals.

Poor cellular communication and compatibility between symbiont partners in opportunistic or heterologous symbionts can elicit host immune responses [88] and adversely affect host performance [29]. Reduced communication and host-symbiont compatibility, therefore, may explain why shifts in symbiont communities are host specific and are generally limited to corals living near limits of their environmental tolerance [23]. Nevertheless, different capacities for Symbiodiniaceae to assimilate carbon and nitrogen in support of host growth [11, 14, 25, 29] appear to also extend to influences on host metabolism and resource allocation [31, 87], with one possible outcome being slower rates of production benefiting symbiont growth and favoring opportunism. Identifying the metabolic tradeoffs from hosting different Symbiodiniaceae communities requires further study, but may prove to be mechanisms by which symbionts benefit while imparting a metabolic cost to the coral host.

*Montipora capitata* tissue (host, symbionts) and skeletal δ^13^C values declined as light decreased, in agreement with increased carbon isotope fractionation as carbon fixation rates decline in low-light environments [45, 61]. During periods of high coral photosynthesis the internal carbon pool becomes enriched in ^13^C (higher δ^13^C values) as dissolved inorganic carbon enriched in ^12^C is preferentially fixed [81]. As a result, the discrimination (i.e., isotope fractionation) against heavy isotopes is reduced and δ^13^C values of photosynthetic products increase [89]. Conversely, reductions in carbon demand/photosynthesis allow for greater isotope discrimination and overall lower δ^13^C values in photosynthetic products and the internal carbon pool. In our study of *M. capitata*, we observed spatiotemporal changes in δ^13^C values to correspond between the host and symbiont, resulting in limited relative differences in carbon isotope values (i.e., δ^13^C_H-S_), a commonly applied metric for greater heterotrophy (δ^13^C_H-S_ values < 0) relative to autotrophy (δ^13^C_H-S_ values > 0) [45–47]. In fact, δ^13^C_H-S_ values became more positive as depth increased, which is opposite to the expected positive correlations between depth and coral heterotrophic nutrition [45]. Considering δ^13^C_H-S_ would be expected to decline as symbiont densities are reduced in stressed corals, we further explored the relationship between δ^13^C_H-S_ and symbiont densities. Again, we find unexpected results: δ^13^C_H-S_ generally increases and becomes more positive in corals as symbiont densities decline (Fig. S9). This relationship may reflect optimal symbiont densities that maximize net photosynthesis and reduce cell shading in healthy corals. Nevertheless, these findings do not support greater heterotrophy in corals in response to light attenuation or reductions in symbiont densities and cast doubt on the effectiveness of using δ^13^C_H-S_ as a proxy for greater heterotrophic nutrition in healthy, non-bleached corals as well as those undergoing symbiont loss due to environmental stress.

Similar disagreements in using δ^13^C_H-S_ values to infer depth-dependent changes in coral nutrition in other studies have been clarified using compound-specific isotope analyses. For instance, *Stylophora pistillata* and *Favia favus* showed no change in δ^13^C_H-S_ across depths (<60 m) in the Red Sea. However, examining the δ^13^C values in lipids between host and symbionts suggested an increase in heterotrophic carbon usage by *S. pistillata* in lipid synthesis below 20 m but consistently low δ^13^C-lipid values in *F. favus* across depths, suggesting high and invariable heterotrophic contributions to lipid biosynthesis in *F. favus*[48]. In bleached and post-bleaching recovered *M. capitata* and *Porites compressa* δ^13^C values showed poor relationships with bleaching history and did not suggest a greater capacity for heterotrophic feeding; instead δ^13^C values were best explained by an isotope mass balance accounting for differences in proteins:lipids:carbohydrates in coral tissues [33]. It is therefore important for uncertainties in isotope values to be further explored in symbiotic mixotrophic organisms and for caution to be applied in the interpretation of these data to infer trophic plasticity. In the present study, changes in host and symbiont δ^13^C values appear to instead be an effect of the interaction between light-availability and symbiont community, in agreement with rapid internal cycling and a shared carbon source of the symbiont partners ([49, 61]). While *M. capitata* biomass and C:N was not influenced by symbiont types or environmental conditions, it is important for future studies to also consider the role of Symbiodiniaceae on coral metabolite profiles [31, 90, 91] which may influence tissue biochemical composition and isotope values [33, 35, 92, 93].

Skeletal carbonate δ^13^C values (i.e., δ^13^C_Sk_) are expected to become more negative as P:R decline [94] or as an effect of greater metabolic fractionation and increased respiratory-derived carbon with low δ^13^C values used in biomineralization [95]. However, *M. capitata* showed relatively small differences in δ^13^C_Sk_ and δ^13^C_H-S_ values across light environments and seasons, suggesting a dominance of autotrophic sources and the maintenance of nutrient recycling between partners. Corals also did not show a decrease in δ^15^N values with depth, as would be predicted as photosynthesis becomes light-limited or if corals are feeding on a prey source with low δ^15^N values [96]. Considering the δ^15^N values of heterotrophic sources in seawater (Fig. S4), corals and their symbionts most resemble the δ^15^N values of dissolved inorganic nitrogen (3.8–4.9‰ [33]) and not plankton end-members. Therefore, *M. capitata* appears to have maintained high rates of photosynthesis (or P:R) as to not show systematic decreases in δ^15^N or ^13^C_Sk_ values and does not show evidence for increased heterotrophy with depth. Predator ^15^N-enrichment factors typically observed in food webs (~3.5 ‰) appear absent or substantially attenuated in corals. The cause for this attenuated trophic enrichment may be nitrogen recycling, wherein symbionts uptake host excreta with low δ^15^N values (i.e., ^14^NH_4_) for amino acid synthesis and translocate a greater proportion of ^14^N-products to the host, effectively lowering δ^15^N values for the holobiont [62]. However, a degree of predator enrichment would still be expected as coral predators and their symbiont retain “heavy” isotope products and exchange “light” isotope products, unless internally recycled nitrogen dominates the host’s nitrogen budget. Alternatively, the isotopic signal originating in prey capture may be overwhelmed by internal and external DIN uptake and nitrogen recycling by Symbiodiniaceae. Further experimental measurements of isotopic compositions of nitrogen sources and downstream products in host and symbiont tissues (i.e., amino acids) may provide a way to clarify heterotrophic influences on coral isotope values (for carbon, see ref. [97]).

## Conclusion

Our analyses of *M. capitata*, therefore, provide three central findings. First, symbiont communities are capable of influencing the isotope values of the holobiont and this fact should be considered in the interpretation and sampling of corals across ecological gradients where symbiont species are partitioned. Second, proxies for assessing greater contributions of heterotrophy relative to autotrophy are more complex than previously recognized [48] and variance originating in host and symbiont δ^13^C values—including proxies for nutritional plasticity (i.e., δ^13^C_H-S_)—may be influenced by physiological factors (i.e., symbiont densities, photoacclimation) unrelated to prey capture. Finally, more nuanced approaches are needed to disentangle the influences of environmental and biological effects on coral carbon and nitrogen isotope values, including compound specific analyses of fatty acids and amino acids [5, 47, 48, 93] and accounting for shifts in biomass composition [33]. Therefore, our findings reinforce the conclusion that facultative shifts in heterotrophic nutrition where they occur are species-specific [48, 98, 99] and context-dependent, perhaps limited to more extreme physiological conditions or to geographic locations favoring mixotrophy (i.e., high near-shore productivity [47]). Further tests of symbiont community effects on isotope values and the energetic consequences of flexible symbiont associations in reef corals should be prioritized in future studies [100].

## Supplementary information

Supplemental materials
