# Supplementary material for: Divergent symbiont communities determine the physiology and nutrition of a reef coral across a light-availability gradient
Source: ISME J. 2020 Jan 3;14(4):945–58. doi: 10.1038/s41396-019-0570-1 (PMC7082336; doi:10.1038/s41396-019-0570-1)
Supplement: Supplementary file 1 — Supplemental materials [file 41396_2019_570_MOESM1_ESM.pdf]

Divergent symbiont communities determine the physiology and nutrition of a reef coral across a light-availability gradient

Christopher B. Wall*, Mario Kaluhiokalani, Brian N. Popp, Megan J. Donahue, Ruth D. Gates

*Corresponding author: cbwall@hawaii.edu

Supplemental Materials

Supplemental materials and methods

Light attenuation coefficients

Light loggers were cross-calibrated using a LI-1400 quantum meter (Li-Cor, Lincoln, USA) attached to a cosine LI-192 underwater quantum sensor. At each site, DLI and depth for loggers at <1 m and 8 m was relativized to the logger at 2 m (i.e., $\Delta DLI = DLI_{2m} - DLI_d$ and $\Delta depth = depth_{2m} - depth_d$). The log(ΔDLI) was analyzed in a no-intercept linear model with the predictor *Δdepth* as a continuous numeric variable. Model coefficients were saved and represented site-specific kd_x. We estimated the seasonal DLI for each sampled colony by calculating the mean DLI at 2 m for summer months (June, July, August) and winter months (November, December, January), and then adjusted for colony-specific depths using site-specific attenuation coefficients, following a modified Beer-Lambert equation for light attenuation in water:

$$Ez_d = Ez_{2m}^{-kd_x*(\Delta depth)}$$

where Ez_d is DLI in mol photons m^{-2} d^{-1} at depth *d* in meters, Ez_{2m} is the mean seasonal DLI at 2 m depth, kd_x is the site-specific attenuation coefficient, and *Δdepth* is the difference in depth at 2 m and depth *d*. To determine the light-at-depth for each coral colony where fragments were

collected, the depth of each colony and the time of collection was noted to correct for changes in tidal height. Final depths were corrected to mean seawater height using NOAA tide data at 6-min intervals for Moku o Lo‘e (Station ID: 1612480) from CO-OPS API in a custom *R* code (Innis et al. 2018).

Dissolved nutrients and plankton sampling

Seawater dissolved nutrients and the isotope values of plankton (i.e., isotope end-members or heterotrophic food sources) were sampled 10 August and 19 December 2016 and used to account for site and/or seasonal differences in nutrient loading and end-members across the study. Molar concentrations (µmol L^{-1}) of ammonium (NH_4^+), nitrate+nitrite (NO_3^- + NO_2^- or N+N), phosphate (PO_4^{3-}) and silicate ($Si(OH)_4$) were analyzed using a Seal Analytical AA3 HR nutrient autoanalyzer at the University of Hawai‘i at Mānoa SOEST Lab for Analytical Biochemistry. Plankton and suspended particles were sampled at the four locations where corals were collected, along with two additional locations in central Kāne‘ohe Bay (21°27'28.7"N, 157°49'37.5"W, and 21°27'35.2"N, 157°49'23.7"W). At each location, plankton was sampled by pooling a vertical (<10 m) and surface horizontal plankton tows (63 µm mesh); visible debris or plant materials were removed and plankton were size-fractioned with nylon mesh in two size classes: 100 – 243 µm and >243 µm. Additionally, seawater samples (10 L) collected at 3 m depth were fractioned with nylon mesh into three size classes: <10 µm, 10 – 100 µm, <243 µm. All samples were filtered onto GF/F filters (0.7 µm) using a vacuum pump at low pressure, rinsed with ddH_2O, and dried at 60 °C overnight. Plankton samples were removed from filters and ground to a powder with mortar and pestle; seawater fractioned materials were left on the GF/F filter, which was subsampled for carbon and nitrogen isotope analysis.

Photopigment analysis

Photopigments (chlorophyll *a* and c_2) were quantified by centrifuging an aliquot of the tissue slurry to isolate symbiont cells (13,000 *g* × 3 min), re-suspending the pellet in 100% acetone, and extracting pigments at -4 °C for 24 h in darkness. Chlorophyll concentrations were measured on a spectrophotometer using a glass 96-well plate at 630 nm and 663 nm and quantified using equations for dinoflagellates (Jeffrey and Humphrey 1975).

Stable isotope analysis

The coral tissue slurry was filtered to remove carbonates (20 µm nylon mesh) (Maier et al. 2010) and the host and symbionts were separated by centrifugation (2000 *g* × 3 min) with filtered seawater rinses (0.2 µm) (Muscatine et al. 1989). Tissues were lyophilized, ground to a powder, and packed in tin capsules for analysis. Analyses of tissue carbon and nitrogen isotope compositions were conducted using a Costech elemental combustion system coupled to a Thermo-Finnigan Delta Plus XP Isotope Ratio Mass Spectrometer (IRMS) at the University of Hawaiʻi at Mānoa SOEST Biochemical Stable Isotope Facility. Plots of sample C:N and $\delta^{13}C$ were explored to test for effects of skeletal carbonates on sample isotope values, and results confirmed filtration was effective in minimizing any effects of carbonate contamination (data not shown). Sample analytical precision ($\delta^{13}C$ and $\delta^{15}N$) was <0.2 ‰ as determined by analysis of laboratory reference materials – glycine and an in-house tuna white muscle tissue – run before and after every 10 coral/algae samples. Glycine standards were previously analyzed using international reference materials and verified with inter-lab comparisons. Standards had known

values of +11.36 ‰ $\delta^{15}N$, -36.49 ‰ $\delta^{13}C$ (glycine) and +14.14 ‰ $\delta^{15}N$, -16.15 ‰ $\delta^{13}C$ (tuna). Coral/algae technical replicates deviating by <0.1 ‰.

Coral skeleton samples were collected by shaving the uppermost layers of the coral skeleton (ca. 2 mm) using a Dremel tool equipped with a diamond-tip, followed by grinding samples to a powder (Rodrigues and Grottoli 2006). Skeletal carbon ($\delta^{13}C_{Sk}$) and oxygen ($\delta^{18}O$) isotope values were measured in ca. 80 µg of skeleton acidified (100% orthophosphoric acid) under vacuum (90 °C) in a common acid bath system. Released CO_2 was analyzed by a GVI Optima Stable Isotope Ratio Mass Spectrometer by the Dr. Howard Spero Laboratory at the University of California at Davis. Laboratory carbonate reference materials and technical replicates deviated by 0.02‰ and <0.2‰ for oxygen and carbon isotope values, respectively. Estimates for carbon and oxygen isotope equilibrium ($\delta^{13}C_{eq}$ and $\delta^{18}O_{eq}$, respectively) for skeletal aragonite were estimated using Kāne'ohe Bay seawater $\delta^{13}C_{eq}$ values of +2.82‰ ($\delta^{13}C_{DIC}$ values of +0.12‰ [analyzed 2006 and 2007]) and an average $\delta^{18}O_{eq}$ value of -1.24‰ for the range of temperature seen in Kāne'ohe Bay (23.0 – 28.0 °C) was estimate ($\delta^{13}C_{seawater}$ of +0.4 ‰ [SMOW]) (Schoepf et al. 2014). A 0.33 slope was applied to isotope equilibrium to plot a kinetic isotope effect (KIE) line, reflecting the simultaneous depletion in heavy isotopes of oxygen and carbon during kinetic and metabolic isotope effects, respectively (McConnaughey 2003).

Symbiodiniaceae communities were quantified by extracting DNA from whole coral fragments or tissue slurry using DNA buffer (0.4 M NaCl, 0.05 M EDTA) with 1 and 2% (w/v) sodium dodecyl sulfate, respectively, following a modified CTAB-chloroform protocol (Cunning et al.

2016; dx.doi.org/10.17504/protocols.io.dyq7vv). Two qPCR reactions (10 µl) were run for each coral sample using a StepOnePlus platform (Applied Biosystems) set to 40 cycles, internal cycle baseline of 3 – 15, and a relative fluorescence (ΔR_n) threshold of 0.01. Symbiont genera present in only one technical replicate were considered absent. In each sample, relative symbiont abundance (i.e., C:D ratio) was determined from amplification threshold cycles (C_T) for *Cladocopium* and *Durusdinium* (i.e., C_T^C, C_T^D) according to the formula C:D = 2^(C_T^C - C_T^D). Gene locus copy number and fluorescence intensity were used to normalize symbiont-specific C_T values (Cunning et al. 2016).

Supplemental results

Nitrogen isotope and C:N values

The nitrogen isotope composition of the coral host ($\delta^{15}N_H$) increased as light declined (p = 0.045) but did not change across seasons or symbiont communities ($p \geq 0.293$) (Figure S9). Symbiont algae $\delta^{15}N$ values marginally increased in D-colonies relative to C-colonies in the winter months (0.3 ‰) (p = 0.017) (Figure S9). The difference between host and symbiont nitrogen isotope values ($\delta^{15}N_{H-S}$) increased as light declined (p = 0.018) and was equivalent among all colonies during summer (ca. +0.4 ‰), but averaged +0.6 ‰ (C-colonies) and +0.3 ‰ (D-colonies) in winter according to symbiont community (p <0.001) (Figure S9). Molar ratios of carbon:nitrogen (C:N) in host and symbionts showed no significant effects ($p \geq 0.134$) (Figure S10). Location explained a large portion of variance for $\delta^{15}N_H$ (75%) and $\delta^{15}N_S$ (80%) models but less (<20%) in $\delta^{15}N_{H-S}$ and C:N models (Figure S5).

References

Cunning R, Ritson-Williams R, Gates RD. Patterns of bleaching and recovery of *Montipora capitata* in Kāneʻohe Bay, Hawaiʻi, USA. Mar Ecol Prog Ser 2016; 551: 131–139.

Innis T, Cunning R, Ritson-Williams R, Wall CB, Gates RD. Coral color and depth drive symbiosis ecology of *Montipora capitata* in Kāneʻohe Bay, Oʻahu, Hawaiʻi. Coral Reefs 2018; 37: 423–430.

Jeffrey SW, Humphrey GF. New spectrophotometric equations for determining chlorophylls a, b, c1 and c2 in higher plants, algae and natural phytoplankton. Biochem Physiol Pflanz 1975; 167: 191–194.

Maier C, Weinbauer MG, Pätzold J. Stable isotopes reveal limitations in C and N assimilation in the Caribbean reef corals *Madracis auretenra*, *M. carmabi* and *M. formosa*. Mar Ecol Prog Ser 2010; 412: 103–112.

McConnaughey TA. Sub-equilibrium oxygen-18 and carbon-13 levels in biological carbonates: carbonate and kinetic models. Coral Reefs 2003; **22**: 316–327.

Muscatine L, Porter JW, Kaplan IR. Resource partitioning by reef corals as determined from stable isotope composition: I. $\delta^{13}C$ of zooxanthellae and animal tissue vs depth. Mar Biol 1989; 100: 185–193.

Rodrigues LJ, Grottoli AG. Calcification rate and the stable carbon, oxygen, and nitrogen isotopes in the skeleton, host tissue, and zooxanthellae of bleached and recovering Hawaiian corals. Geochim Cosmochim Acta 2006; 70: 2781–2789.

Schoepf V, Levas SJ, Rodrigues LJ, McBride MO, Aschaffenburg MD, Matsui Y, *et al*. Kinetic and metabolic isotope effects in coral skeletal carbon isotopes: A re-evaluation using experimental coral bleaching as a case study. Geochim Cosmochim Acta 2014; 146: 164–178.

Supplemental tables and figures

Supplemental Table S1. Model analysis of environmental variables (daily light availability, dissolved inorganic nutrients, and isotopic values of size fractioned plankton and particles) at four reefs in Kāne'ohe Bay*.

Environmental variable	*Effect*	*SS*	*df*	*F*	*p*
[a]Daily light integral (DLI)†	Location	4378.754	3,530	134.674	**<0.001**
(mol photons m^{-2} d^{-1})	Season	1040.907	1,210	96.043	**<0.001**
	Location × Season	490.862	3,531	15.097	**<0.001**
[b]Dissolved inorganic nutrients					
phosphate	Location	0.005	4	1.218	0.426
(PO_4^{3-} µmol L^{-1})	Season	0.009	1	8.182	**0.046**
	Residual	0.004	4		
ammonium	Location	0.090	4	5.696	0.060
(NH_4^+ µmol L^{-1})	Season	1.325	1	336.712	**<0.001**
	Residual	0.018	4		
nitrate + nitrite	Location	0.488	4	294.012	**<0.001**
(NO_3^- + NO_2^- µmol L^{-1})	Season	0.067	1	162.024	**<0.001**
	Residual	0.002	4		
silicate	Location	21.495	4	1.629	0.324
($Si(OH)_4$ µmol L^{-1})	Season	3.612	1	1.095	0.354
	Residual	13.194	4		
[b]Size fractioned plankton and particles					
carbon isotope values ($\delta^{13}C$)	Location	17.926	5	1.342	0.263
	Season	7.921	1	2.965	0.914
	Size fraction	76.419	4	7.150	**0.001**
	Residual	130.920	49		
nitrogen isotope values ($\delta^{15}N$)	Location	2.326	5	1.729	0.146
	Season	1.094	1	4.065	**0.049**
	Size fraction	30.377	4	28.234	**<0.001**
	Residual	13.180	49		

* Model outputs are linear models with Type II analysis of variance tables, except for †, where model output is linear mixed effect model with Date as a random effect. *SS* = sum of squares; *df* = degrees of freedom; for †, *df* is degrees of freedom in numerator and denominator; bold *p* values represent significant effects ($p < 0.05$).
Data collection periods are indicated by superscripts (*a-c*):
[a] 10 June 2016 – 12 January 2017
[b] 20 August 2016 and 19 December 2016

Supplemental Table S2. Statistical analysis of *Montipora capitata* physiology from four locations in Kāne‘ohe Bay along a light-availability gradient in summer and winter.

Dependent variable	*Effect*	*SS*	*df*	*F*	*p*
biomass (mg cm^{-2})	Season	0.159	1,116	0.003	0.959
	Light	4.358	1,108	0.072	0.789
	Symbiont	162.958	1,116	2.690	0.104
symbionts (cells cm^{-2})	Season	0.008	1,112	0.133	0.716
	Light	0.392	1,115	6.866	**0.010**
	Symbiont	3.133	1,113	54.830	**<0.001**
	Season × Light	0.481	1,113	8.411	**0.004**
total chlorophyll ($a + c_2$ µg cm^{-2})	Season	45.581	1,114	25.545	**<0.001**
	Light	16.042	1,110	8.990	**0.004**
	Symbiont	31.622	1,115	17.721	**<0.001**
	Season × Symbiont	9.718	1,113	5.411	**0.022**
chlorophyll per cell ($a + c_2$ pg symbiont $cell^{-1}$)	Season	0.147	1,114	2.825	0.096
	Light	2.387	1,116	43.977	**<0.001**
	Symbiont	5.125	1,114	98.716	**<0.001**

Season = summer or winter, *Light* = light at depth of collection, *Symbiont* = *Cladocopium* spp. (formerly clade C) or *Durusdinium glynnii* (formerly clade D) dominated symbiont community. *SS* = sum of squares; *df* = degrees of freedom in the numerator and denominator; bold *p* values represent significant effects ($p < 0.05$).

Supplemental Table S3. Statistical analysis of *Montipora capitata* tissue isotope values from four locations in Kāne'ohe Bay along a light-availability gradient in summer and winter.

Dependent variable	*Effect*	*SS*	*df*	*F*	*p*
$\delta^{13}C_H$	Season	1.032	1,111	1.398	0.240
	Light	23.638	1.114	32.005	**<0.001**
	Symbiont	27.456	1.112	37.174	**<0.001**
	Season × Light	0.170	1,112	0.231	0.632
	Season × Symbiont	3.523	1,112	4.770	**0.031**
$\delta^{13}C_S$	Season	0.002	1,113	0.002	0.962
	Light	35.816	1,115	44.529	**<0.001**
	Symbiont	12.375	1,113	15.386	**<0.001**
	Season × Symbiont	8.757	1,113	10.887	**0.001**
$\delta^{13}C_{H\text{-}S}$	Season	1.320	1,111	9.931	**0.002**
	Light	0.360	1,113	2.712	0.102
	Symbiont	2.291	1,113	17.243	**<0.001**
	Season × Light	0.574	1,113	4.322	**0.040**
	Season × Symbiont	0.590	1,112	4.441	**0.037**
$\delta^{13}C_{Sk}$	Season	4.888	1,115	6.961	**0.009**
	Light	0.155	1,115	0.221	0.639
	Symbiont	0.002	1,115	0.003	0.953
$\delta^{15}N_H$	Season	0.109	1,113	1.132	0.290
	Light	0.418	1,114	4.327	**0.040**
	Symbiont	0.038	1,113	0.392	0.532
$\delta^{15}N_S$	Season	0.001	1,112	0.014	0.907
	Light	0.002	1,113	0.022	0.882
	Symbiont	0.790	1,112	7.241	**0.008**
	Season × Symbiont	0.644	1,112	5.903	**0.017**
$\delta^{15}N_{H\text{-}S}$	Season	0.104	1,114	1.849	0.177
	Light	0.323	1,115	5.767	**0.018**
	Symbiont	0.538	1,114	9.588	**0.002**
	Season × Symbiont	0.963	1,113	17.173	**<0.001**
$C{:}N_H$	Season	0.369×10^{-3}	1,115	0.070	0.792
	Light	0.004	1,114	0.703	0.403
	Symbiont	0.001	1,115	0.155	0.695
$C{:}N_S$	Season	0.021	1,115	2.281	0.134
	Light	0.344×10^{-3}	1,115	0.037	0.847
	Symbiont	0.016×10^{-3}	1,115	0.002	0.967

Season = summer or winter, *Light* = light at depth of collection, *Symbiont* = *Cladocopium* (formerly clade C) or *Durusdinium* (formerly clade D) dominated symbiont community. *SS* = sum of squares; *df* = degrees of freedom in numerator and denominator; bold *p* values represent significant effects ($p < 0.05$). *Subscripts* indicate coral host (H), symbiont algae (S), or their relative difference (H-S), and skeletal carbonates (Sk).

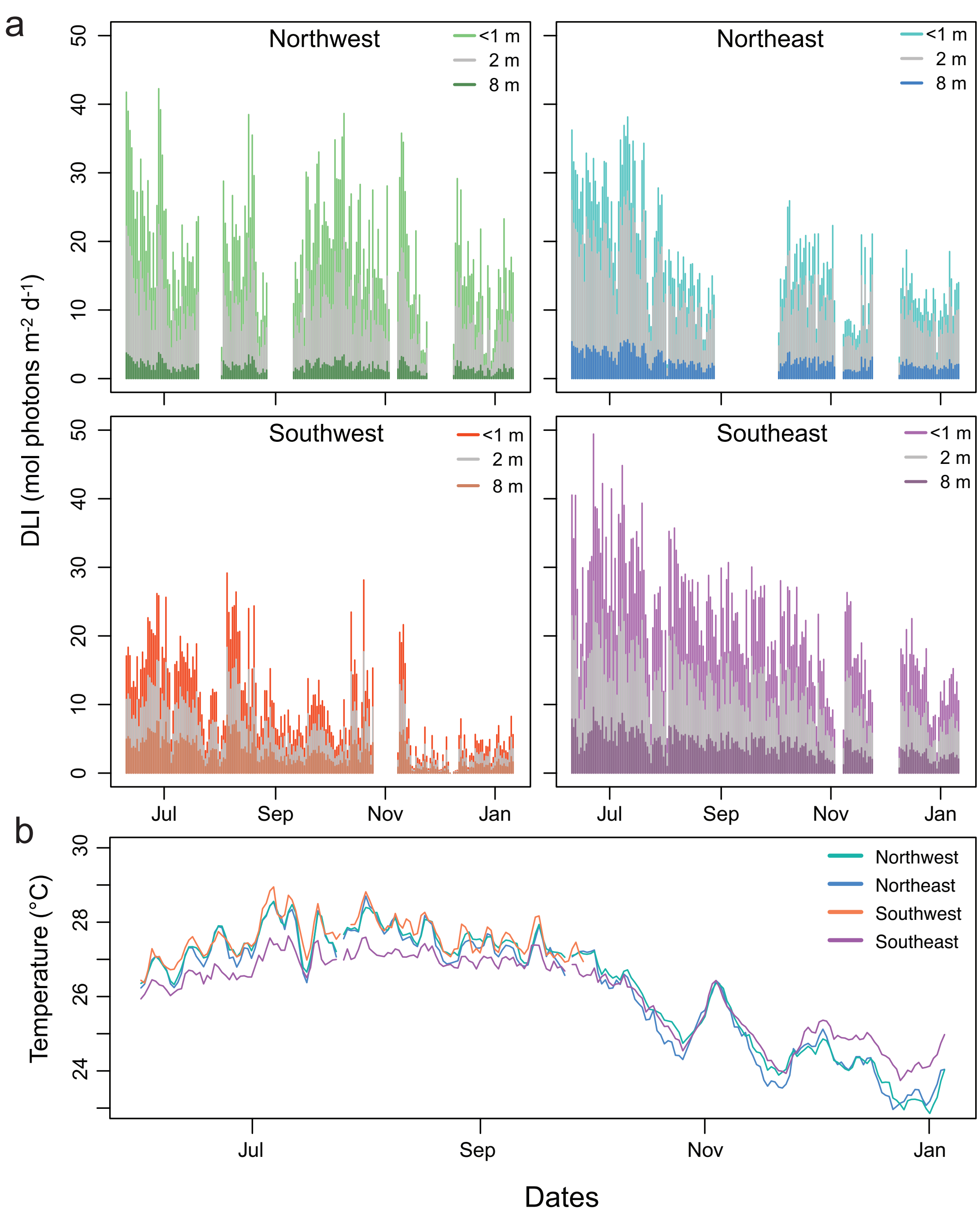

Supplemental Figure S1. (**a**) Light availability (daily light interval [DLI]) and (**b**) daily mean temperature recorded at 2 m-depth at four Kāne‘ohe Bay reefs from June 2016 – January 2017. DLI values are based on measured values at 2 m depth and calculating light at <1 m and 8 m according to a modified Beer-Lambert equation for light attenuation in water.

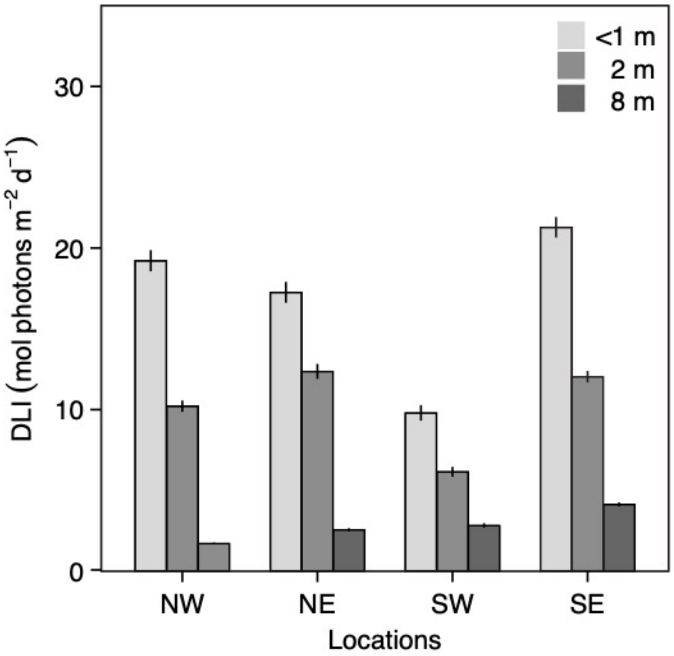

Supplemental Figure S2. Daily light integral (DLI) at four reef locations where corals were collected, averaged over the study period (10 June 2016 – 12 January 2017) at <1 m, 2 m and 8 m depth. Values are mean ± SE (n = 163 – 202).

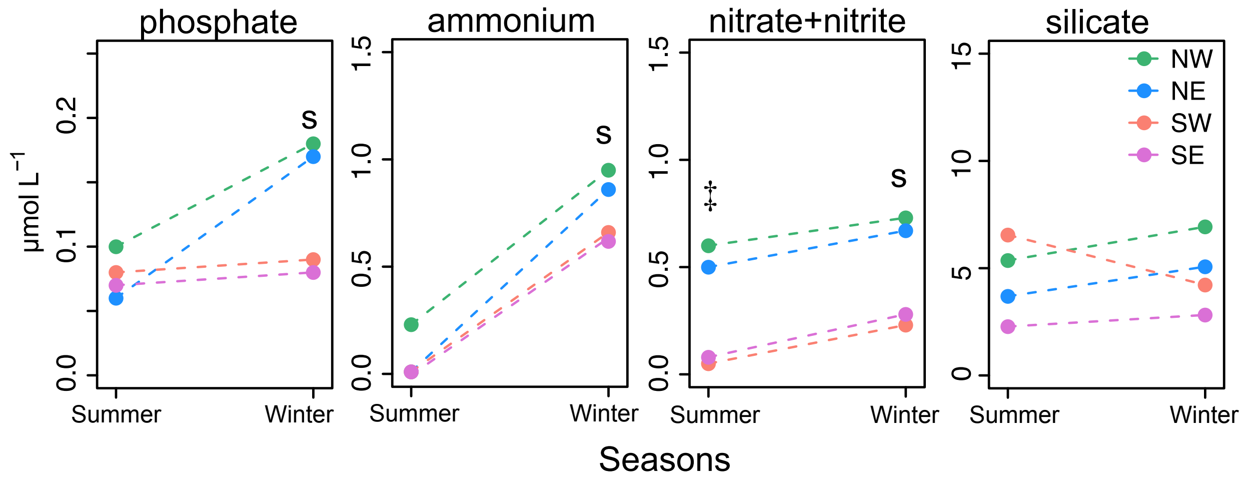

Supplemental Figure S3. Molar concentrations of the dissolved inorganic nutrients (μmol L^{-1}) phosphate (PO_4^{3-}), ammonium (NH_4^+), nitrate+nitrite (NO_3^- + NO_2^- or N+N) and silicate ($Si(OH)_4$) in seawater (points, n = 1) collected during two sampling periods in summer and winter 2016 from four reef locations, described in *Figure 1*. *Symbols* indicate significant differences ($p < 0.05$) between seasons (*s*) and among locations (‡).

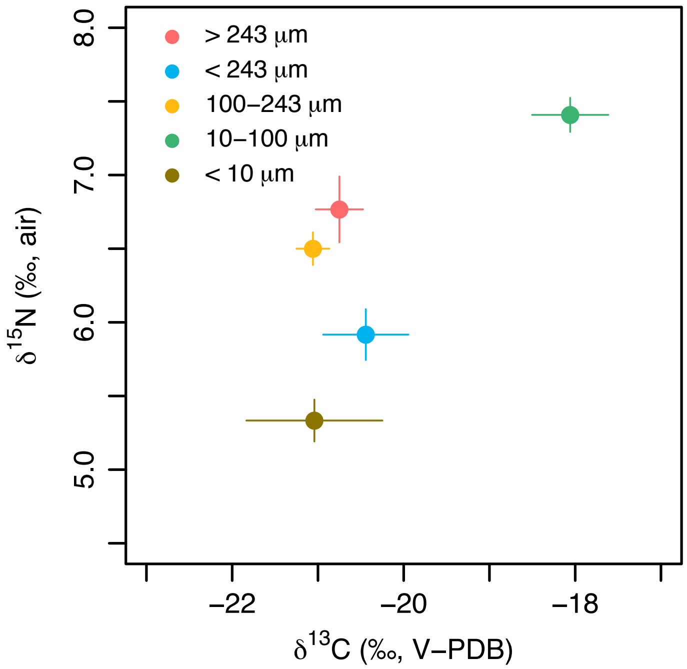

Supplemental Figure S4. Size fractioned organic materials and plankton in seawater. Values are mean ± SE (*n* = 12) in permil (‰) relative to standards for carbon (Vienna-Pee Dee Belemnite: V-PDB) and nitrogen (air).

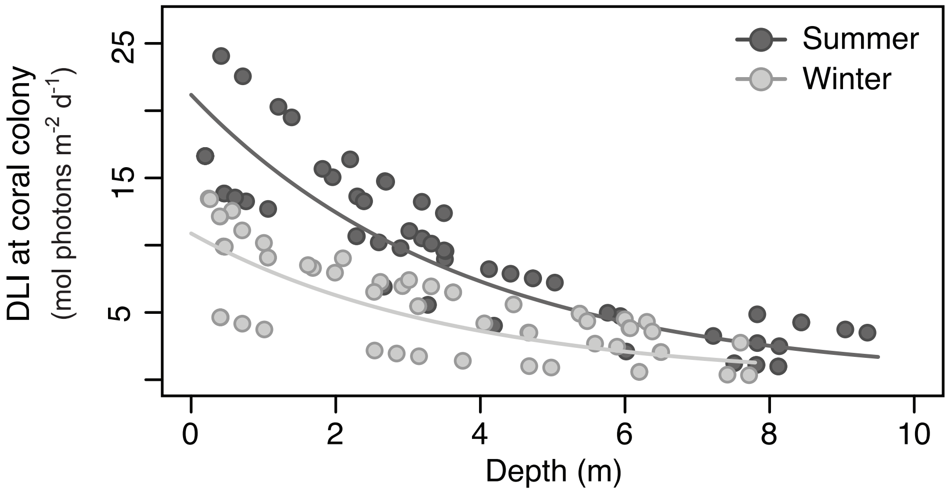

Supplemental Figure S5. The daily light integral (DLI) and the depth (m) where *Montipora capitata* coral fragments were collected during two periods (summer and winter) in 2016 from Kāne‘ohe Bay, O‘ahu, Hawai‘i. Solid lines represent model fit to log(DLI) and depth relationship in each season.

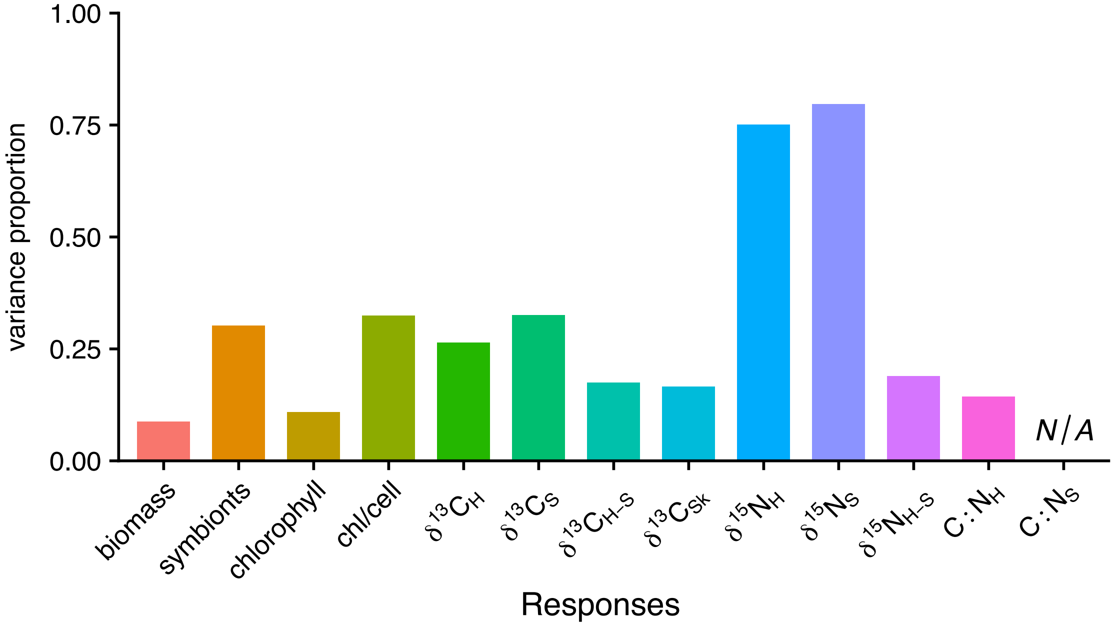

Supplemental Figure S6. Proportion of linear mixed effect model variance explained by the random effects of *Location* for each response metric. *N/A* represents models where the proportion of variance accounted for by random effects was not different from zero.

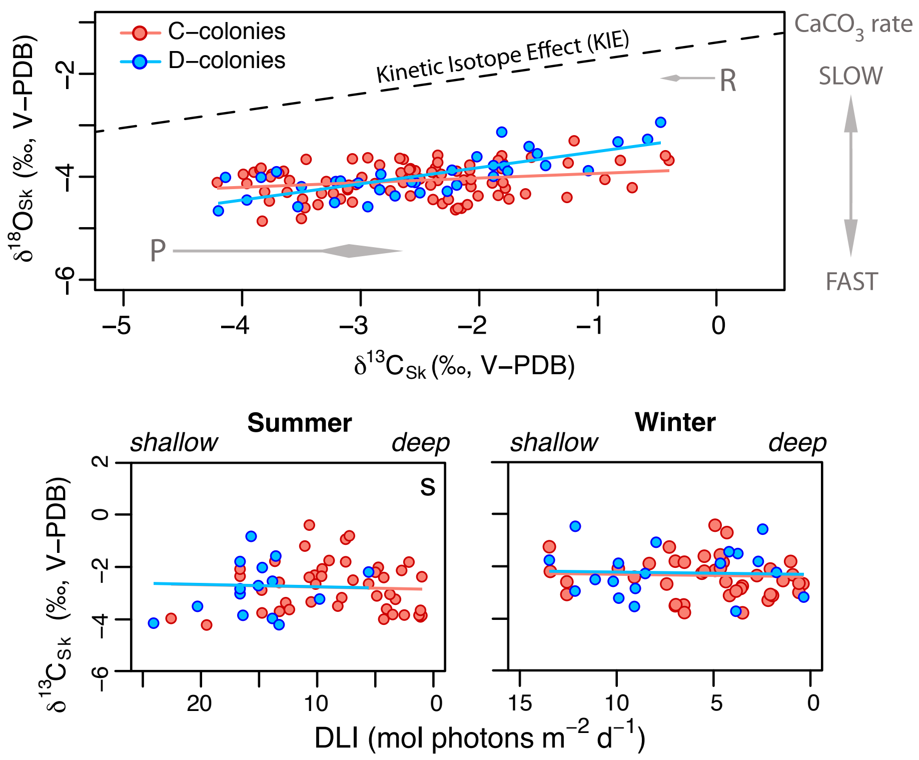

Supplemental Figure S7. Stable isotope values skeletal carbonates from *Montipora capitata* colonies dominated by C (*Cladocopium* spp.) or D (*Durusdinium* spp.) symbionts. (*Top*) The relationship between coral skeletal carbonate oxygen ($\delta^{18}O_{Sk}$) and carbon stable isotope values ($\delta^{13}C_{Sk}$), and (*bottom*) changes in $\delta^{13}C_{Sk}$ values in response to light availability across seasons. Letters *P* and *R* represent carbon isotope offsets from metabolic effects of photosynthesis and respiration, respectively. *Slow* and *fast* refer to skeletal growth effects ($CaCO_3$ rate) on $\delta^{18}O_{Sk}$; Kinetic Isotope Effect (KIE) is the line where kinetic isotope effects occur, departing from seawater isotopic equilibrium (approx. -1.24 ‰ $\delta^{18}O$ and 2.85 ‰ $\delta^{13}C$). Values are permil (‰) relative to standards for carbon and oxygen (Vienna-Pee Dee Belemnite: V-PDB). Solid lines represent linear mixed effect model fits. *Letters* (*s*) indicate significant differences ($p < 0.05$) between seasons.

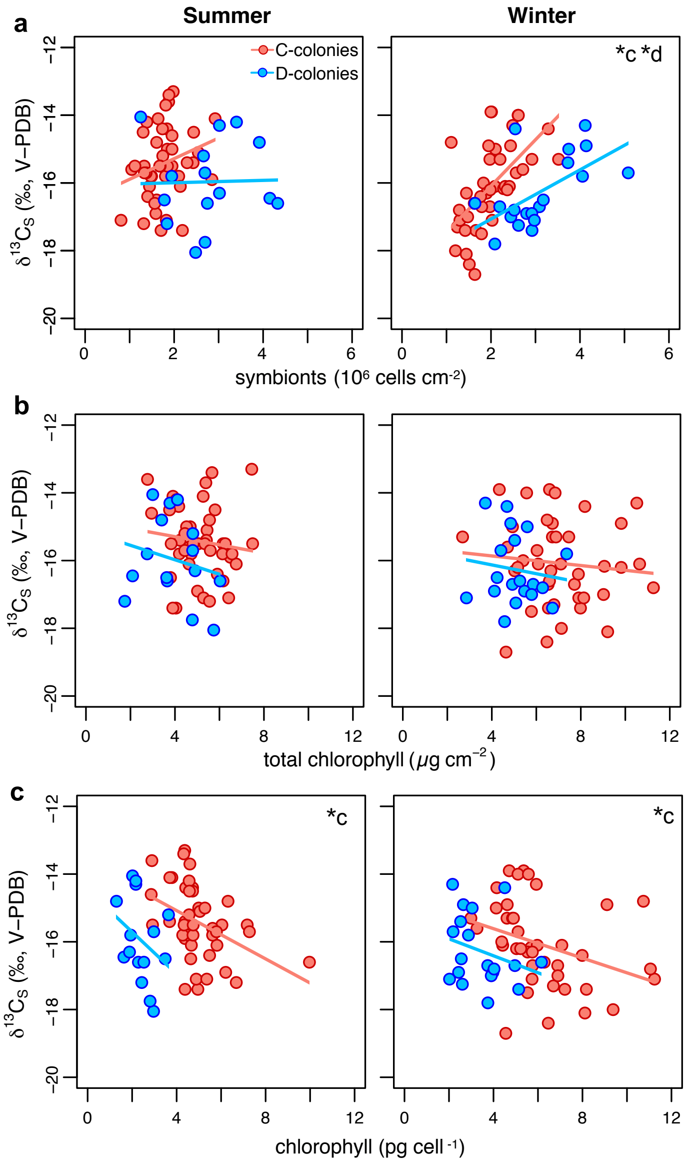

Supplemental Figure S8. The relationship between symbiont isotope values ($\delta^{13}C_S$) and (**a**) symbiont densities, (**b**) total chlorophyll, and (**c**) chlorophyll per symbiont cell for *Montipora capitata* colonies dominated by C (*Cladocopium* spp.) or D (*Durusdinium* spp.) symbionts collected in summer (*left*) and winter (*right*). Solid lines represent linear model fits. *Asterisk-letters* represent significant relationship ($p < 0.05$) for C- or D-colonies (**c* or **d*, respectively).

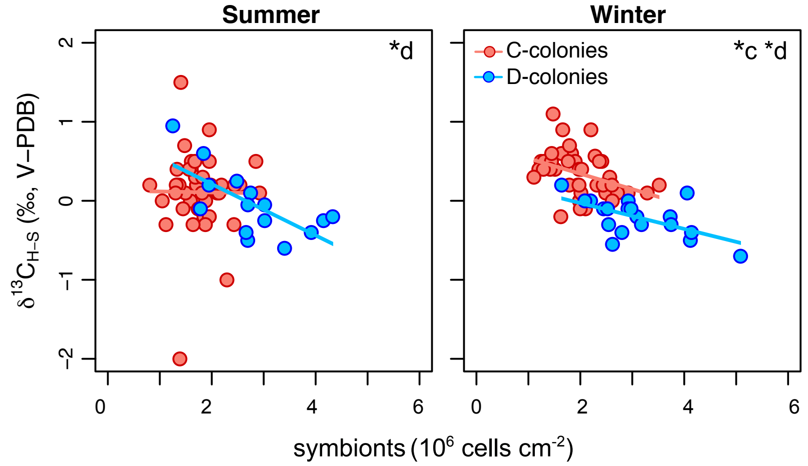

Supplemental Figure S9. The relationship between the relative differences in host and symbiont carbon isotope values ($\delta^{13}C_{H\text{-}S}$) and symbiont densities for *Montipora capitata* colonies dominated by C (*Cladocopium* spp.) or D (*Durusdinium* spp.) symbionts collected in summer (*left*) and winter (*right*). Solid lines represent linear model fits. *Asterisk-letters* represent significant relationship ($p < 0.05$) for C- or D-colonies (**c* or **d*, respectively).

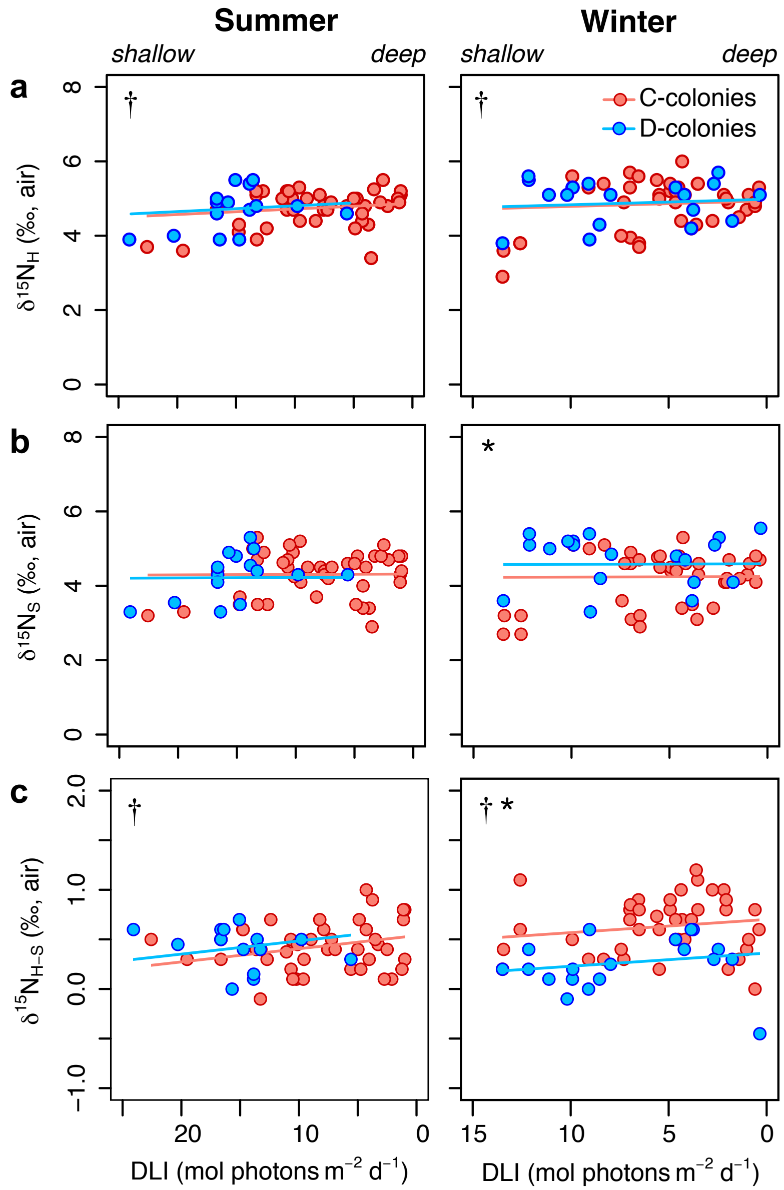

Supplemental Figure S10. Nitrogen stable isotope values for *Montipora capitata* colonies dominated by C (*Cladocopium* spp.) or D (*Durusdinium* spp.) symbionts collected in summer (*left*) and winter (*right*). Values are (**a**) coral host ($\delta^{15}N_H$) (**b**) symbiont algae ($\delta^{15}N_S$) and (**c**) their relative difference ($\delta^{15}N_{H\text{-}S}$) in permil (‰) relative to nitrogen standards (air). Lines represent linear mixed effect model fits. *Symbols* indicate significant differences ($p < 0.05$) between symbiont communities (*) or in response to light (†).

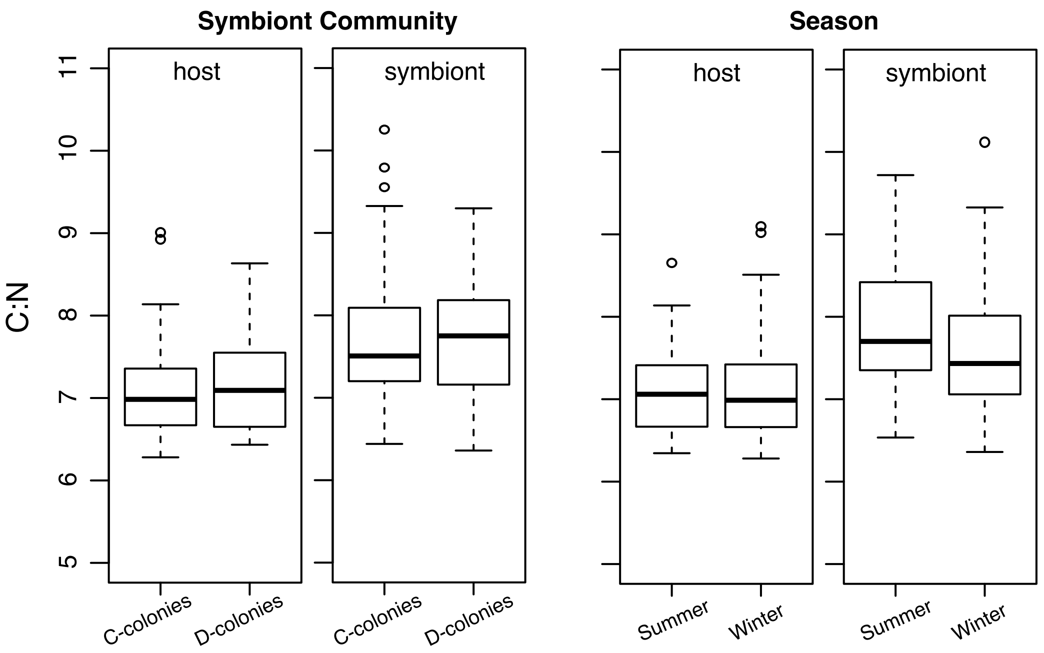

Supplemental Figure S11. Biomass molar carbon:nitrogen (C:N) ratios in host and symbiont tissues as a function of symbiont community (C-colonies *vs.* D-colonies) and season (summer *vs.* winter).
